# The Determinants of Adherence to Public Health and Social Measures Against COVID-19 Among the General Population in South Korea: National Survey Study

**DOI:** 10.2196/35784

**Published:** 2023-01-17

**Authors:** Hye Chong Hong, Hyeonkyeong Lee, Suk Jeong Lee, Chang Park, Mikyung Lee

**Affiliations:** 1 Red Cross College of Nursing Chung-Ang University Seoul Republic of Korea; 2 College of Nursing Mo-Im Kim Nursing Research Institute Yonsei University Seoul Republic of Korea; 3 Department of Population Health Nursing Science University of Illinois Chicago Chicago, IL United States

**Keywords:** COVID-19, preventive measures, health literacy, trust, national survey, Tobit regression

## Abstract

**Background:**

The COVID-19 pandemic has created devastating health, social, economic, and political effects that will have long-lasting impacts. Public health efforts to reduce the spread of COVID-19 are the priority of national policies for responding to the pandemic globally. Public health and social measures (PHSMs) have been shown to be effective when used alone or in combination with other measures, reducing the risk of spreading COVID-19. However, there is insufficient evidence on the status of compliance with PHSMs in the general population for the prevention of COVID-19 in public areas, including Korea.

**Objective:**

The aim of this study was to assess levels of compliance with the recommended PHSMs against SARS-CoV-2 infection and their predictors among the general population by using national data.

**Methods:**

This study was a secondary data analysis of the National Survey of Infectious Disease Preventive Behaviors in Community, which was conducted by the Korea Centers for Disease Control and Prevention Agency (KDCA) between October 12 and October 30, 2020. The primary study was cross-sectional, using stratified sampling via an adjusted proportional allocation method to select representative samples and ensure the stability of samples. The data were collected through phone interviews conducted by trained enumerators using a structured questionnaire. PHSM adherence was measured using a 10-item comprehensive infectious disease prevention behavior (CIDPB) scale, and each sociocognitive factor, including perceived susceptibility to SARS-CoV-2 infection, perceived severity of SARS-CoV-2 infection, perceived confidence in performing preventive behaviors related to COVID-19, information comprehension ability, and trust in information from the KDCA, was measured. A total of 4003 participants were included in the final analysis. Tobit regression and a decision tree analysis were performed to identify the predictors of preventive measures and the target groups for intervention.

**Results:**

We discovered that women scored 1.34 points higher on the CIDPB scale than men (*P*<.001). Compared to the group aged 19 to 29 years, those aged 50 to 59 years and those older than 60 years scored 1.89 and 2.48 points higher on the CIDPB scale (*P*<.001), respectively. The perceived severity of infection, confidence in preventive behaviors, information comprehension ability, and trust in information from the KDCA were significant positive determinants of CIDPBs (*P*<.001). The perceived susceptibility to infection showed a significant negative relationship with CIDPBs (*P*<.001).

**Conclusions:**

Female sex, older age, lower income, and sociocognitive factors were found to be significant determinants of adhering to PHSMs. The findings suggest the need for tailored interventions for target groups; specifically, the age group that was the most active at work indicated the highest potential to spread infection. Adequate public health education and health communication for promoting adherence to PHSMs should be emphasized, and behavior change strategies for those with low perceived confidence in performing PHSMs should be prioritized.

## Introduction

The COVID-19 pandemic is a global health crisis. The pandemic has created devastating health, social, economic, and political effects that will have long-lasting impacts in many parts of the world [1,2]. SARS-CoV-2 is a novel virus causing an outbreak of mild to severe respiratory diseases. COVID-19 has affected millions of people around the world. As of September 2021, a total of 219 million cases and over 4.55 million deaths were reported. Korea is no exception to this devastating crisis. More than 276,000 total cases and 2367 deaths were reported as of September 2021 [3]. Although COVID-19 vaccines are now available in many countries, vaccination does not guarantee complete protection from contracting COVID-19, and it will take a while to achieve herd immunity, as a substantial proportion of the population needs to be vaccinated [4]. Research is still ongoing to find out how strong the vaccines are against COVID-19, how effectively they would reduce transmission, and how long the effects of vaccination last. Until herd immunity is achieved, preventive measures should still be important means for reducing the transmission of COVID-19 and protecting ourselves from contracting an evolving infectious disease in the future [4].

Public health efforts to reduce the spread of COVID-19 are still the priority of national policies for responding to the pandemic globally. Most countries have announced public health and social measures (PHSMs) against COVID-19. PHSMs include personal protective measures, such as handwashing and mask wearing in public areas; environmental measures, such as disinfection and ventilation; surveillance and response measures, such as contact tracing, isolation, and quarantine; physical distancing measures, such as limiting the size of gatherings and maintaining distance in public areas; and limitations on international travel [5]. These protective measures have been shown to be effective when used alone or in combination with several measures, reducing the risk of spreading COVID-19 [6-8]. The Korea Centers for Disease Control and Prevention Agency (KDCA) has also announced guidelines for PHSMs that are similar to WHO guidelines [9]. Nevertheless, there is insufficient evidence on the status of compliance with PHSMs in the general population for the prevention of COVID-19 in public areas, including Korea.

Researchers have reported the disparities in compliance with PHSMs by socioeconomic groups. In general, being male, having a lower education and a lower income, living in rural areas, and being younger are associated with performing fewer preventive behaviors. [10-13]. Mistrust toward the health care system and health literacy are other important factors that contribute to the disparities in PHSM compliance. People who do not trust the health care system are less likely to follow recommendations if the recommendations are from the system that they do not trust, especially when these recommendations are inconsistent and difficult to understand [14]. An effective and rapid response to COVID-19 requires the public to follow recommended behaviors, and public health information plays a crucial role [15]. However, people vary in terms of their abilities to obtain and understand health information and use services to make informed decisions (ie, health literacy) [16], and conflicting messages about PHSMs for COVID-19 have created confusion in the public, which has caused delays in following PHSMs [17]. In fact, McCaffery et al [15] reported disparities in COVID-19–related knowledge, attitudes, and behaviors by health literacy level. However, more studies are needed to confirm the relationships among mistrust in the health care system, health literacy, and preventive behaviors in the new era of the COVID-19 pandemic.

Health beliefs are closely related to health behaviors. According to the Rosenstock Health Belief Model [18], perceived susceptibility (beliefs about acquiring a disease), perceived severity (beliefs about the seriousness of risk), and self-efficacy (confidence in the ability to control a situation to achieve a goal) are important concepts that could influence people’s decisions to engage in health behaviors. Although sparse, recent research revealed that health beliefs are significant predictors of COVID-19 preventive health behaviors [13,19,20]. Public health efforts reduce the spread of COVID-19 both globally and within countries, including Korea. Examining the factors that influence preventive health behaviors would provide opportunities to target interventions toward those who are in need. To date, there is a lack of knowledge about factors related to PHSMs against COVID-19. Thus, the purpose of this study was to assess levels of compliance with the recommended preventive measures against SARS-CoV-2 infection and their predictors among the general population by using national data.

## Methods

### Data Source and Sample

This study was a secondary data analysis of the National Survey of Infectious Disease Preventive Behaviors in Community, which was conducted by the KDCA between October 12 and October 30, 2020. The survey was developed based on the following steps: (1) a literature review on previous studies related to infectious diseases and associated preventive behaviors; (2) in-depth interviews with experts in infectious diseases on preventive behaviors and their predictors; (3) the pooling of items based on the literature review and in-depth interviews with experts; (4) the development of a pilot questionnaire based on the pooled items; (4) content validity index calculation with 11 experts; and (5) a pilot test with 20 adults on the difficulty, relevance, and comprehensiveness of the questionnaire. The survey included data on adherence to PHSMs, socioeconomic characteristics, experiences of respiratory and intestinal infectious disease symptoms, experiences of isolation or hospitalization due to SARS-CoV-2 infection, perceived susceptibility to COVID-19, perceived severity of COVID-19, confidence in performing preventive behaviors, COVID-19–related information comprehension ability, and trust in information from the KDCA.

A representative sample of the population aged 19 to 69 was stratified based on sex (male and female) and age groups (19-29 years, 30-39 years, 40-49 years, 50-59 years, and 60-69 years) in 17 cities or provinces nationwide. Stratified sampling via an adjusted proportional allocation method was used to select representative samples and to ensure the stability of samples in 17 cities or provinces. First, 50 people from each city or province were allocated. Second, a proportional allocation method was used to reduce or increase the sample size based on the number of people residing in each city or province. Therefore, the number of people selected from each city or province ranged from 25 to 1050. To increase the representativeness of the sample, a quota ratio was used to allocate samples based on sex and age groups; thus, the characteristics of the samples selected were consistent with those of the population. Trained professional enumerators conducted computer-assisted telephone interviews with participants, using random digital dialing, between October 12 and October 30, 2020. The interviews were stopped when the target proportion was reached. A total of 421,428 phone calls were made, and 5025 individuals completed telephone interviews. We included 4003 participants in this study after carefully reviewing and excluding participants with missing data on the dependent variable.

### Ethical Considerations

This study was a secondary analysis of a pre-existing data set, and ethical review and approval were exempted for this study by the institutional review board of the first author’s university (Chung-Ang University). The primary study was conducted by the KDCA, and informed consent was obtained prior to the phone interviews. The primary data contained anonymous information.

### Measures

#### Adherence to PHSMs

Adherence to PHSMs was measured by using a comprehensive infectious disease prevention behavior (CIDPB) scale. The preliminary items were developed based on the review of literature published within the last 5 years in PubMed, Embase, Cochrane Library, and the Research Information Sharing Service on prevention behaviors for infectious diseases; existing instruments for COVID-19 prevention behaviors [21]; and the COVID-19 prevention guidelines from the Centers for Disease Control in the United States and Korea. The 10 items, including handwashing, cough etiquette, and social distancing, were constructed after a review by experts and content validity testing. Examples of items include (1) washing hands thoroughly for 30 seconds with soap and running water, (2) covering the nose and mouth with sleeves when coughing or sneezing, and (3) maintaining a 2-m (at least 1 m) distance from others. Each item was answered on a 4-point Likert scale (1=always; 2=most of the time; 3=sometimes; 4=never) and was reverse coded. The participants who answered “not applicable” or “don’t know” and those who refused to answer were excluded from the analysis. The total score ranged from 4 to 40, with higher scores indicating the practicing of more preventive behaviors. The Cronbach α was 0.76 (95% CI 0.747-0.770), supporting the instrument’s internal consistency.

#### Sociocognitive Factors

The participants were asked to answer questions related to perceived susceptibility to SARS-CoV-2 infection, perceived severity of SARS-CoV-2 infection, and perceived confidence in performing preventive behaviors related to SARS-CoV-2 infection, using a single question for each factor. Each question was answered on a 7-point Likert scale (1=not at all; 7=very likely), with a higher score indicating higher perceived susceptibility to SARS-CoV-2 infection, perceived severity of SARS-CoV-2 infection, perceived confidence in performing preventive behaviors, and information comprehension ability with regard to COVID-19. Information comprehension ability was assessed by asking the following question: “How easy is it for you to understand the various information given during COVID-19?” Participants were asked to answer on a 7-point Likert scale (1=very hard; 7=very easy), with a higher score indicating the easier comprehension of COVID-19 information.

Trust in information from the KDCA was assessed by asking the following question: “How much trust in information provided by KDCA?” Participants were asked to respond on a 10-point Likert scale (1=never trust; 10=very trust), with a higher score indicating more trust in information from the KDCA.

#### Demographic Variables

The demographic variables included in this study were age, sex (ie, male or female), educational level (ie, middle school or lower, high school, or college or above), monthly household income (ie, less than US $5000 or greater than US $5000), and living areas (urban or rural areas). Further, 8 metropolitan cities and cities in 9 provinces were categorized as urban areas, and the villages in the provinces were categorized as rural areas. Age was categorized as 19 to 29 years, 30 to 39 years, 40 to 49 years, 50 to 59 years, or ≥60 years.

### Statistical Analysis

We analyzed the data by using STATA 15.1 (StataCorp LLC), SPSS 25 (IBM Corp), and R (R Foundation for Statistical Computing). A weighted statistical analysis was performed to increase the representativeness of the data. Descriptive statistics, such as means, SDs, frequencies, and percentages, were used to describe the participants. To examine the relationships that demographics, sociocognitive factors, and trust in information from the KDCA had with CIDPBs, the Mann-Whitney *U* test and the Kruskal-Wallis H test were done, as the CIDPBs were not normally distributed. A post hoc analysis was done by using Bonferroni corrections [22]. We then performed Tobit regression to examine the effects of demographics, sociocognitive factors, and trust in information from the KDCA on CIDPBs. A Tobit regression model, which is also called a *censored regression model*, was performed to estimate linear relationships among variables when the dependent variable was censored to either the left or right [23]. The dependent variable in this study (the CIDPBs) was skewed to the right, and this analysis allows one to specify a threshold to censor the regression. Further, a decision tree analysis was conducted by using the classification and regression tree (CART) technique introduced by Breiman et al [24] via the SPSS statistical package. CART analysis is a nonparametric technique for identifying each predictor in order to identify the most important predictor at each step when a sample is divided into 2 mutually exclusive and homogeneous subpopulations. The starting group is referred to as the *root*, each split is referred to as a *branch*, and the data subset resulting from the split is called a *node*, while the terminal nodes are referred to as *leaves* [24]. A single configuration of hyperparameters was chosen to reduce the complexity of CARTs to ensure sensible bias-variance trade-off. The minimum number of cases in the parent node and child node was 100 and 50, respectively. The chosen parameters for growing trees were a maximum tree depth of 5 and a significance threshold of .05 for splitting. The “±1 SE rule” was applied to prune the tree.

## Results

The characteristics of participants are summarized in Table 1. The average age of participants was 45.4 (SD 14.98) years. Of the 4003 participants, 1947 (48.6%) were male. Approximately 57.3% (2265/3953) of participants were educated at a college or higher level. About 58% (1941/3349) earned less than US $5000 per month, and 42% (1408/3349) earned more than US $5000 per month. Most of the participants (3741/4003, 93.5%) resided in the city. Bivariate analyses were performed to examine the association between general characteristics and the CIDPBs (Table 2).

The mean CIDPB scale score was 34.91 (SD 3.74) and ranged from 15 to 40. Sex, age, the level of education, monthly income, and living areas were significantly associated with the CIDPBs. The mean score of male participants was slightly lower than that of female participants (*P*<.001), and the mean score for participants residing in urban areas was lower than that for participants residing in rural areas. The Bonferroni post hoc test revealed that the older age group had more CIDPBs. Specifically, the participants aged 19 to 29 years had the lowest mean score compared to those of other age groups (*P*<.001). The participants aged 30 to 39 years had a lower mean score than those of the participants aged 40 to 49 years (*P*=.001) and the participants aged 50 years and older (*P*<.001). The participants aged 40 to 49 years had a lower mean score than those of the participants aged 50 to 59 years (*P*=.002) and the participants aged 60 years and older (*P*<.001). The participants aged 50 to 59 years had a lower mean score than that of the participants aged 60 years and older (*P*<.001).

**Table 1:** General characteristics (N=4003) of participants in the National Survey of Infectious Disease Preventive Behaviors in Community (conducted on October 2020 in South Korea).

Characteristics		Participants, n (%)
**Sex**		
	Male	1947 (48.6)
	Female	2056 (51.4)
**Age (years)**		
	19-29	737 (18.4)
	30-39	693 (17.3)
	40-49	799 (20)
	50-59	773 (19.3)
	≥60	1001 (25)
**Education**		
	Middle school or lower	350 (8.9)
	High school	1338 (33.8)
	College or higher	2265 (57.3)
**Monthly household income (US $)**		
	<5000	1941 (58)
	≥5000	1408 (42)
**Location** **of residence**		
	Urban	3741 (93.5)
	Rural	262 (6.5)

**Table 2:** Level of compliance by general characteristics (N=4003) of participants in the National Survey of Infectious Disease Preventive Behaviors in Community (conducted on October 2020 in South Korea).

Characteristics			Score, mean (SD)		*z* test		*P* value		Post hoc test^a^
**Sex**					−9.86		<.001		
	Male	34.4 (3.97)						N/A^b^	
	Female	35.5 (3.40)						N/A	
**Age (years)**					263.95		<.001		
	19-29 (A)	33.4 (3.97)						A<B, C, D, E	
	30-39 (B)	34.2 (3.78)						B<C, D, E	
	40-49 (C)	34.8 (3.64)						C<D, E	
	50-59 (D)	35.5 (3.40)						D<E	
	≥60 (E)	36.1 (3.38)							
**Education**					67.56		<.001		
	Middle school or lower (A)	36.3 (3.50)							
	High school (B)	34.8 (3.87)						B<A	
	College or higher (C)	34.7 (3.66)						C<A	
**Monthly household income (US $)**					−4.28		<.001		
	<5000	35.2 (3.66)						N/A	
	≥5000	34.6 (3.69)						N/A	
**Location** **of residence**					−3.09		.002		
	Urban	34.9 (3.75)						N/A	
	Rural	35.6 (3.57)						N/A	

^a^Bonferroni test order.

^b^N/A: not applicable.

A Tobit regression model analysis was performed to identify predictors of CIDPBs (Table 3). The results revealed that the CIDPB score was 1.34 (95% CI 1.09-1.59) points higher in female participants when compared to that of male participants. The participants who were aged between 50 and 59 years scored higher on the CIDPB scale (performed more CIDPBs) by 1.89 (95% CI 1.45*-*2.34) points, and the participants who were older than 60 years scored higher on the CIDPB scale (performed more CIDPBs) by 2.48 points (95% CI 2.02*-*2.93), when compared to those who were aged between 19 and 29 years. The perceived severity of infection, confidence in preventive behaviors, information comprehension ability, and trust in information from the KDCA were statistically significant and positive (*P*<.001). The perceived susceptibility to infection was statistically significant and negative (*P*=.02).

**Figure 1:**
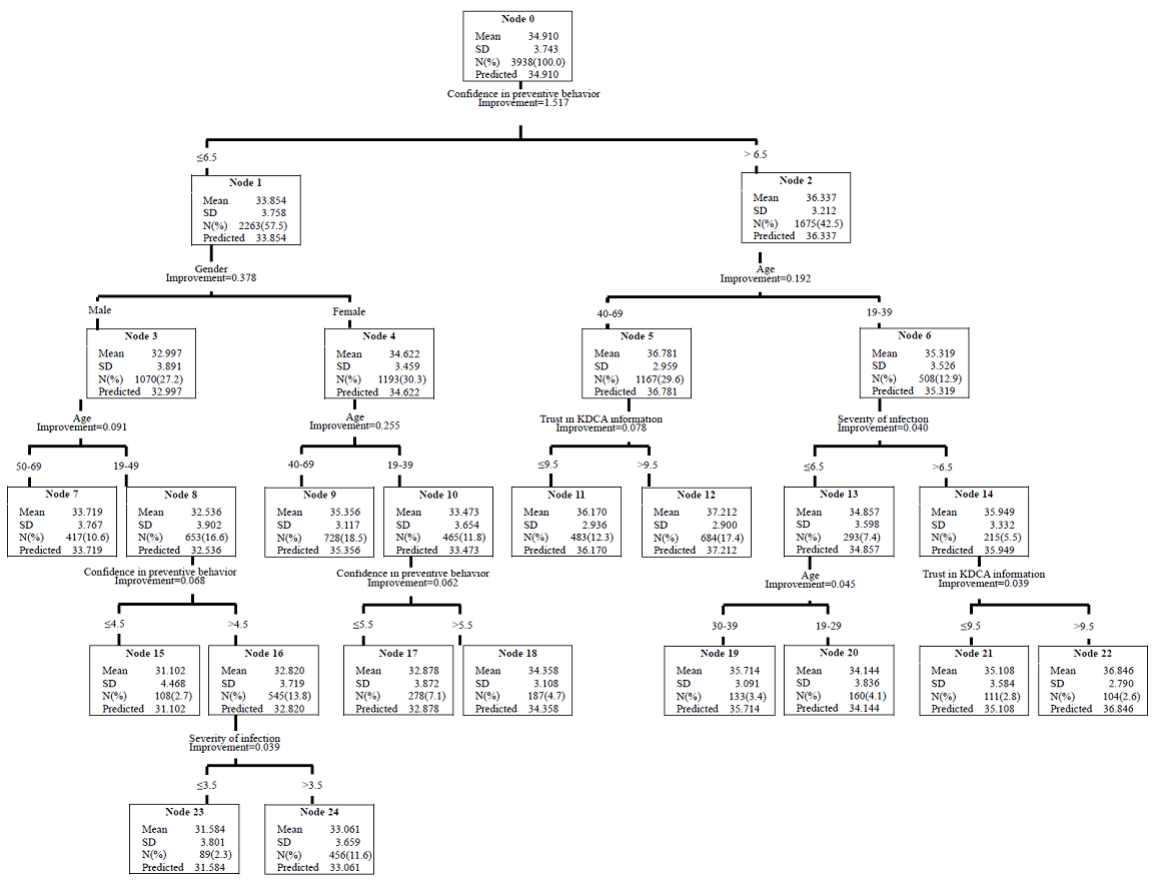
Key factors associated with the comprehensive infectious disease prevention behaviors in the National Survey of Infectious Disease Preventive Behaviors in Community (conducted in October 2020 in South Korea). The classification and regression tree analysis results show relationships with various independent variables. KDCA: Korea Centers for Disease Control and Prevention Agency.

**Figure 2:**
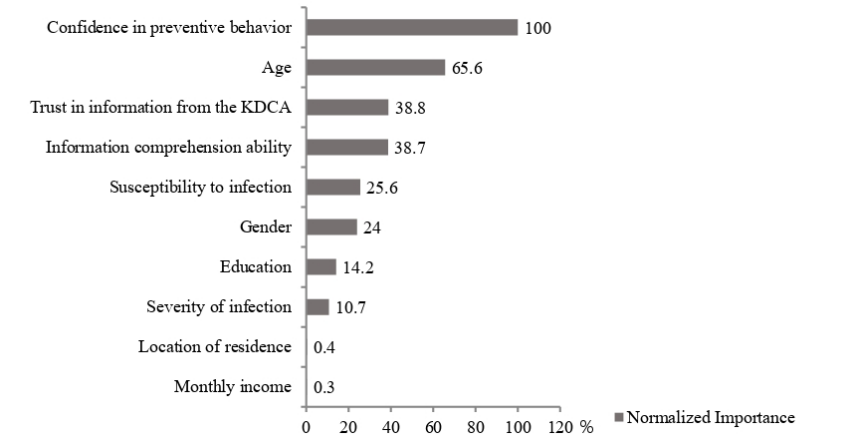
Normalized importance of independent variables based on the classification and regression tree results for the National Survey of Infectious Disease Preventive Behaviors in Community (conducted on October 2020 in South Korea). KDCA: Korea Centers for Disease Control and Prevention Agency.

Figure 1 depicts the decision tree produced by the CART analysis. Perceived confidence in performing preventive behaviors (scores of >6.5) was the first classifying dimension, which was mostly associated with CIDPBs. The results suggested that the participants aged 40 to 69 years with trust in information from the KDCA (scores of >9.5) had higher scores on the CIDPB scale (range 34.91-37.21). Men aged 19 to 49 years with scores of <4.5 for perceived confidence in performing preventive behaviors were the least likely to practice CIDPBs, with CIDPB scale scores ranging from 34.91 to 31.10. Perceived confidence in performing preventive behaviors showed the highest normalized importance among variables for explaining CIDPBs, followed by the age group and trust in information from the KDCA variables (Figure 2).

**Table 3:** Factors associated with preventive behaviors based on a Tobit regression model^a^ in a National Survey of Infectious Disease Preventive Behaviors in Community (conducted on October 2020 in South Korea).

Characteristics		Coefficient (95% CI)	*P* value	
**Sex**				
	Male	Reference		
	Female	1.34 (1.09 to 1.59)	<.001	
**Age (years)**				
	19-29	Reference		
	30-39	0.84 (0.37 to 1.30)	<.001	
	40-49	1.41 (0.96 to 1.85)	<.001	
	50-59	1.89 (1.45 to 2.34)	<.001	
	≥60	2.48 (2.02 to 2.93)	<.001	
**Education**				
	Middle school or lower	Reference		
	High school	−0.35 (−0.90 to 0.21)	.22	
	College or higher	−0.30 (−0.87 to 0.26)	.29	
**Monthly household income (US $)**				
	<5000	Reference		
	≥5000	−0.47 (−0.73 to−0.20)	<.001	
**Location of residence**				
	Urban	Reference		
	Rural	0.38 (−0.07 to 0.83)	.10	
**Sociocognitive factors**				
	Perceived susceptibility to infection	−0.08 (−0.15 to −0.02)	.02	
	Perceived severity of infection	0.14 (0.06 to 0.22)	<.001	
	Perceived confidence in preventive behavior	0.84 (0.72 to 0.97)	<.001	
	Information comprehension ability	0.21 (0.09 to 0.33)	<.001	
	Trust in information from the KDCA^b^	0.22 (0.14 to 0.30)	<.001	

^a^Log pseudo likelihood=−8416.22; *F*_14,3321_=48.79; *P*<.001; pseudo *R*^2^=0.04.

^b^KDCA: Korea Centers for Disease Control and Prevention Agency.

## Discussion

### Principal Findings

The purpose of our study was to assess levels of compliance with the recommended PHSMs against SARS-CoV-2 infection and their predictors among the general population by using national data. There is a need for health care providers and public health officials to know which groups are in need of education on PHSMs. With regard to socioeconomic factors, sex (female), age, monthly income, and sociocognitive factors were significantly associated with more preventive behaviors. Consistent with previous research, female participants were more likely to perform preventive behaviors. During epidemics of infectious diseases, including the influenza, H1N1, and COVID-19 pandemics, it was shown that women are more sensitive to risk and are more motivated to perform health behaviors than men [25-27]. Interestingly, people who were older were more likely to perform preventive behaviors than those who were younger, which is consistent with a study by Raude et al [28]. The possible explanation for this finding is that people who are older may believe that they are more prone to infection and that they will be sicker and experience more complications once they are infected and thus engage in more preventive behaviors [29].

Surprisingly, those with lower incomes were more engaged in PHSMs. A previous study reported that people with higher incomes tend to stay at home more often when compared to those with lower incomes [29] and show higher compliance with preventive behaviors against COVID-19 when compared to those with a low economic status [11]. People with lower incomes may engage in jobs that are unstable and may work in environments that increase the likelihood of infection (eg, environments in which the proximity between people cannot be maintained or confined areas with limited ventilation), which may place them at risk for infection. However, the relationship between income and preventive behavior needs to be examined in future studies, as the exact pathway for why people with lower incomes are more engaged in preventive behaviors is not well established in the literature.

Consistent with previous findings [13,26,30,31], the influence of confidence in the ability to control a situation on health behavior change was found to be a strong determinant of adherence to PHSMs for preventing COVID-19 and the intention to follow preventive behaviors [32]. Moreover, in this study, we attempted to identify target groups that are in the most need of intervention to increase preventive behaviors, and we found that men aged between 19 and 49 years with lower scores for perceived confidence in performing preventive behaviors are in the most need of intervention. As this age group is the most active at work and in daily life, it should be given a higher priority in terms of their potential to spread infection to others. Given that the majority of the participants were employed (2,933/4003, 73%), policies and guidelines encouraging the practice of preventive behaviors and the provision of essential infection control materials should be established at work. In addition, men tended to dismiss preventive actions when compared to women in this study, which is consistent with the findings from a study of 8 countries [33]. Thus, gender perspectives are taken into account when designing education programs to promote preventive behaviors among a target population.

It is well believed that a person with high perceived susceptibility is more likely to take actions to reduce the risk of acquiring a disease. Thus, perceived susceptibility gained importance as a significant psychosocial factor related to COVID-19 preventive behaviors in earlier studies [32,34,35]. However, the temporal relationship between perceived susceptibility and preventive behaviors should be interpreted with caution. In contrast to these previous studies, we found a negative association between perceived susceptibility to infection and preventive behaviors. Since there was a negative association between perceived susceptibility and preventive behaviors, we may interpret the results as follows: those who do not perform preventive behaviors or perform fewer preventive behaviors may believe that they are at more risk of contracting diseases. In addition, as addressed in an earlier study on the use of mobile apps to trace COVID-19 cases [36], considering the relatively high adherence to PHSMs among the majority of participants in this study (34.91/40), another possible reason could be that the better the preventive action, the lower the perceived risk of contracting COVID-19. However, this study was cross-sectional; thus, we cannot assume this causal inference. Longitudinal studies are needed in the future to examine the causal relationship between sociocognitive factors and preventive behaviors.

The more a person believes that a given disease is serious, the more that person will try to reduce the chance of contracting that disease by performing preventive behaviors [37]. Health literacy is the knowledge and competence required to understand and process health information, and it influences individuals to make proper health decisions [16]. In fact, better health literacy, which we defined as information comprehension ability, was associated with more preventive behaviors in our study, which is consistent with previous studies [37,38]. The most important health information includes knowledge about COVID-19, skills for preventive behaviors, and government policies [38,39]. There is a lot of information about COVID-19 released by the media, which includes false information. Good health literacy enables people to differentiate between facts and myths regarding information about COVID-19, which influence people to perform appropriate preventive behaviors [17]. Health messages about COVID-19 are extremely confusing and change rapidly. People who do not trust information released by the health care system and the government are less likely to follow recommended behaviors [14]. In fact, our study results indicate that people with less trust in the information from the KDCA are less likely to perform preventive behaviors. Adequate government action and policy development are needed to ban the release of false information that may cause irrational panic and prevent people from practicing preventive behaviors. Moreover, demographic variables, such as low education and older age, are known to be associated with low health literacy levels [38,40]; thus, people with low educational levels and those who are older need to be targeted for intervention to increase health literacy.

Some limitations should be noted. First, our study was cross-sectional; thus, temporal inferences should be interpreted with caution. Second, the sample size may be small, although we included nationally representative samples by using stratified sampling and a proportional allocation method. Thus, longitudinal studies with larger sample sizes are needed to confirm the causality between preventive behaviors and predictors. Third, the primary study was conducted by using questionnaires; thus, recall bias may be present. Fourth, some of the variables, such as sociocognitive factors, were measured with a single item; therefore, there is a possibility that we did not capture the multidimensional concepts of each variable. In future studies, more reliable and valid instruments that capture all aspects of sociocognitive factors may need to be used. Fifth, we only included individual factors in the analysis. Thus, multilevel analyses may be necessary, as preventive behaviors could be affected by community and national factors. Sixth, this study was implemented before the outbreaks of COVID-19 variants, including the Omicron variants. Therefore, the study results need to be interpreted with caution in the context of the new COVID-19 variants. Lastly, this study did not include variables related to the vaccine awareness, as this study was conducted before the start of national vaccination in South Korea. Further studies are needed to assess the relationships between vaccination and preventive behaviors. Despite these limitations, our findings add new knowledge to previous literature, as we identified predictors of preventive behaviors by using nationally representative samples collected during the COVID-19 pandemic in Korea.

### Conclusion

The purpose of this study was to assess levels of compliance with the recommended preventive measures against SARS-CoV-2 infection and their predictors among the general population by using national data. In addition to identifying predictors, we have provided insights into the specific groups that may need targeted interventions, such as men aged between 19 and 49 years with low perceived confidence in performing preventive behaviors. A theory-driven intervention may need to be carefully designed to meet the needs of the target population, and such an intervention should be delivered at appropriate places, such as workplaces, to reach a large number of people. Moreover, efforts to increase health literacy and trust in the health care system are needed at the community and national levels. However, the lack of a temporal relationship between preventive behaviors and predictors indicates the need for longitudinal studies in the future.

## Abbreviations

CART: classification and regression tree
CIDPB: comprehensive infectious disease prevention behavior
KDCA: Korea Centers for Disease Control and Prevention Agency
PHSM: public health and social measure
